# CogChamps: impact of a project to educate nurses about delirium and improve the quality of care for hospitalized patients with cognitive impairment

**DOI:** 10.1186/s12913-018-3286-4

**Published:** 2018-07-09

**Authors:** Catherine Travers, Amanda Henderson, Frederick Graham, Elizabeth Beattie

**Affiliations:** Queensland University of Technology, Kelvin Grove, QLD Australia

**Keywords:** Dementia, Delirium, Hospitals, Education, Nursing

## Abstract

**Background:**

Achieving sustainable practice changes to ensure best-practice nursing care in acute hospital environments can be challenging and is not well understood. A multi-faceted practice change intervention was implemented in a large Australian hospital to enhance the capacity of the nursing workforce to provide quality care for older patients with cognitive impairment (CI).

**Methods:**

Thirty-four experienced Registered Nurses (RNs) became Cognition Champions (CogChamps), and led practice-change initiatives to improve nursing care for older patients (≥65 years) on six wards in one hospital. The CogChamps received comprehensive education about dementia and the identification, prevention, and management of delirium. Over five months, they were supported to develop and implement ward-specific Action Plans designed to change care practices. Nurse-patient interactions were observed and patient charts were audited prior to the implementation of the plans and regularly throughout, using a purpose built Audit/ Observational tool. Data were also collected at a comparable hospital where there were no CogChamps. Data were analyzed for evidence of practice change.

**Results:**

Observational and audit data were collected for 181 patients (average age = 82.5 years) across the two hospitals. All patients had CI and both cohorts had similar behavioral characteristics requiring a high level of care assistance [e.g. 38% displayed evidence of confusion/disorientation and a majority experienced meal-time difficulty (62–70%)]. While nursing practices were generally the same at both hospitals, some differences were evident (e.g. analgesia use was higher at the control hospital). Following implementation of Action Plans, significant increases in nurses’ assessments of patients’ cognitive functioning (35 to 69%), and administration of analgesia (27 to 48%) were observed at the intervention hospital, although only the improvement in cognitive assessments was maintained at three months follow-up. No other changes in nursing processes were evident.

**Conclusion:**

The CogChamps project demonstrates how RN champions were empowered to educate their colleagues about dementia and delirium resulting in a sustained increase in cognitive assessments by ward nurses. Practice improvements were mostly associated with clearly defined Action Plan tasks and goals and where responsibility for task completion was clearly assigned. These elements appear to be important when implementing practice changes.

**Trial registration:**

Australian Clinical Trials Registration Number: ACTRN 12617000563369. Retrospectively registered.

## Background

Cognitive impairment (CI) including dementia and delirium are common in older patients admitted to acute hospitals. Recent data indicates that between 20 and 25% of patients aged 70 years and older admitted to an acute hospital have dementia [1, 2] while much higher rates are reported in older age groups (up to 47% in patients 90 years and older). Rates for delirium are reported to range from 10 to 31% at admission and from 3 to 29% during the hospital episode [3] while rates of up to 70% have been reported in intensive care units. Both dementia and delirium are associated with a range of adverse outcomes including longer admissions, functional decline, institutionalisation and death [4]. Despite this, and despite delirium often being preventable [5, 6], CI is often not detected in hospital patients.

Thus, improving the care of older hospitalized patients with cognitive impairment (CI) including dementia and delirium, has been recognized as a priority issue by leading healthcare organizations including the Australian Commission on Safety and Quality in Healthcare (ACSQHC) [7], England’s National Health Service [8], and the UK’s Royal College of Nursing [9]. Within Australia, the challenge is being led by the Australian Commission on Safety and Quality in Healthcare (ACSQHC), which has launched the Caring for Cognitive Impairment Campaign [7]. that focuses on improving staff knowledge and care practices to reduce the disproportionate risk of harm in this patient group. Particular emphasis is directed towards the early identification of CI through accurate assessment in order that strategies can be implemented to prevent potential complications, the most frequent of which is delirium [10].

There is, however, limited evidence regarding the most effective way of educating staff and of initiating practice change for older patients with CI in the hospital environment. The few studies that have been undertaken indicate that multi-factorial interventions, together with the use of knowledge translation principles, are more effective than other approaches in promoting practice change and preventing delirium [6, 11]. Accordingly, we developed an interventional protocol that upskilled RN champions to improve the capacity of an acute-care nursing workforce to recognize patients with CI and provide high quality care. The intervention (described in full elsewhere [12]), adopted a “distributed leadership” approach to improving clinical practice in which self-nominated Cognition Champions (CogChamps) facilitated the collective social education of ward staff. The CogChamps were provided in-depth education on evidence-based assessment and multi-factorial management approaches to caring for cognitively impaired patients in hospital and were encouraged to develop “Action Plans for Practice Change” pertinent to their individual ward settings. The overall approach used constructs of evidence, context and facilitation essential for successful practice change [13], and the implementation plan addressed multiple factors that are detailed in Table 1.

**Table 1:** Factors addressed in the CogChamps implementation plan

Evidence	
➢ There is sound evidence that non-pharmacological strategies can effectively prevent delirium in many at-risk patients [6] and reduce Behavioral and Psychological Symptoms of Dementia [37];	
➢ Nurses’ reported preference for accessing evidence-based knowledge by engaging with local clinical experts rather than with online guidelines and text, was acknowledged [38, 39], and responded to by equipping and supporting ward based Registered Nurses (RNs) to undertake the role of Cognition Champion and provide a readily accessible knowledge source;	
Context	
➢ Executive level support was obtained – key hospital staff including the Nurse Unit Managers of the wards, and medical consultants of the wards involved were members of the project’s Steering Committee and supported the project;	
➢ The policy context – implementation of the project coincided with the launch of ACSQHC’s Caring for Cognitive Impairment Campaign [7], to which the hospital made a public commitment (the commitment is publicly acknowledged on the Campaign website);	
➢ The hospital context – the project was implemented in an environment in which a sound foundation for caring for patients with CI had been established through the prior implementation of several initiatives led by the hospital’s dementia and delirium specialist [FG: Clinical Nurse Consultant – Dementia and delirium]. For details, see [12].	
➢ The ward context – Cognition Champions (CogChamps) were assisted to develop ward specific Action Plans to promote their engagement in the project and ensure interventions were tailored to address each ward’s specific requirements;	
➢ Adequate resources were available for the project – external funding allowed project staff to allocate sufficient time to assist hospital staff to implement and evaluate activities;	
Facilitation	
➢ Facilitation played a central role in promoting practice change through mentorship, direct support and the provision of feedback to CogChamps regarding their progress;	

This paper describes the activities undertaken by the CogChamps to improve patient care, and reports patient outcomes following the project’s implementation. Details of the educational approach adopted by the CogChamps and nurses’ ability to recognize delirium have been published in a companion paper [14].

### Aims of the intervention

The aims were to:

- educate and empower experienced hospital nurses to lead practice changes on their wards, in order to
- improve the care of hospitalized patients with CI by implementing best practices, in particular care processes for delirium prevention, identification and management [15, 16].

## Methods

### Procedure

The methods have been comprehensively described in the protocol paper [12], and hence are only briefly outlined here. Registered Nurses (RNs) with two or more years’ clinical experience were invited to become Cognition Champions (CogChamps) if they demonstrated (a) a specific interest in dementia and delirium, or alternatively, (b) displayed suitable leadership skills. The role of the CogChamp was to champion best practice care for older patients with CI (dementia and delirium) at the local ward level. Approximately equal numbers (four – six) of CogChamps were identified and recruited from each of six hospital wards in the Intervention Hospital (IH). Over two full days of workshops, the CogChamps were provided with comprehensive dementia and delirium education (Workshop One) and skills development around leadership and change management (Workshop Two). In Workshop Two, CogChamps worked in ward-specific groups to identify priorities for improving the care of patients with CI on their wards. This culminated in six ward-specific Action Plans outlining achievable actions that the CogChamps could implement to improve patient care on their wards.

The Research Team assisted the CogChamps to refine their Action Plans in the month following Workshop Two so that all Plans included a minimum of three specific actions with measurable outcomes and associated timeframes. They were also supported to establish sound communication processes amongst themselves (e.g. use of a communications book and email to schedule meetings and send project updates), and encouraged to include project updates at regular ward meetings. To further facilitate progress, a CogChamps leader was identified for each ward who assumed overall responsibility for coordinating activity.

Members of the research team assisted the CogChamps to implemented their Action plans by meeting with one or more CogChamp(s) from each ward weekly (face to face and/email) to assess progress, provide feedback, and support them over the five month implementation phase. The research team also assisted the CogChamps to source evidence-based resources and assisted the them to analyze any data they collected. Although the CogChamps were not allocated any protected time for project implementation, they received support from dedicated project facilitators. These were CogChamps who were employed as facilitators for a limited time in a full-time capacity (funding was provided by the Research grant). Three CogChamps were seconded to undertake the Facilitator CogChamp role. One worked across the two surgical wards for six weeks while the role was shared by two CogChamps who worked across the medical wards for seven weeks (one worked for three and one for four weeks). As they were relatively inexperienced or ‘novice’ facilitators, they received mentorship and ongoing support from a member of the Research Team (AH), very experienced in implementing practice change initiatives. Key project activities and associated timelines are summarized in Table 2.

**Table 2:** CogChamps: Key activities and timelines

1	Baseline Data Collection
	➢ Data were collected pre-intervention to establish baseline measurements,
	➢ Research Nurses collected data in the study wards at the Intervention and Control hospitals using the Audit/ Observational tool (see Methods),
	➢ Timeframe - 2 weeks,
2	CogChamps: Education and Empowerment
	➢ CogChamps were recruited from 6 hospital wards (4–6 per ward) at the Intervention hospital,
	➢ CogChamps received education about dementia and delirium (1 full-day Workshop)^a^ and leadership and change management skills (1 full-day Workshop);
	➢ CogChamps commenced development of ward-specific Action Plans – 6 were developed,
	➢ CogChamps were assisted to refine their Action Plans in month following Workshop Two to ensure plans included three specific actions that could be implemented over 5–6 months with measurable outcomes and associated timeframes,
	➢ Time frame – approximately 2 months,
3	Data Collection 2 (Post CogChamps education)
	➢ To assess any impact of educating CogChamps on study outcomes,
	➢ Methods were as for Baseline,
4	Implementation Phase
	➢ CogChamps received support to implement their Action Plans,
	➢ Time frame – 5 months
5	Data Collection 3 (Post implementation of Action Plans)
	➢ To assess any impact of the implementation of Action Plans,
	➢ Methods were as for Baseline and Data Collection 2,
6	Follow-Up Data Collection
	➢ Undertaken 3 months following the withdrawal of the Research team to assess the sustainability of changes over the longer term,
	➢ Methods were as for Baseline and Data Collections 2 and 3,

^a^Comprehensive details are provided in [12]

### The setting

Implementation occurred across six wards (four medical and two surgical) of a large tertiary referral hospital - the Intervention Hospital (IH), located in in South-East Queensland, Australia. General medical and surgical wards were chosen as both settings have a high prevalence of patients with CI and we were interested to see if cultural workplace differences influenced outcomes. All wards were staffed by a regular cohort of 30–40 nurses. The impact and effectiveness of the interventions at the IH were assessed by comparing the uptake of best practices, and outcomes at this site with another comparable site, located nearby (approximately 10 km away). Two wards (one medical, one surgical) in this hospital served as control wards (Control Hospital; CH), and the two hospitals are comparable insofar as nursing staff have been exposed to the same educational modules for dementia and delirium as IH nurses and they have similar patient profiles.

### Outcome measures

Project outcomes included:

- Increased nurses’ knowledge of CI and self-confidence in nursing patients with CI;
- Increased number of nurses in the IH who are proficient in assessing and documenting dementia and delirium;
- Increased numbers of patients assessed for CI at admission to hospital (IH);
- Improved outcomes of best practices for nursing older patients with CI (e.g., improved pain management, nutrition & hydration, patient mobilization) [4, 15], and
- Reduced adverse outcomes for older patient with CI in hospital (i.e., falls, antipsychotic use).

Outcomes (a) and (b) are reported briefly below (see ‘implementation of Action Plans’) and in full in the companion paper [14], while outcomes (c) – (e) are reported herein.

### The Room & Chart Audit/ observation tool

A Chart Audit/ Observational tool was developed to capture data regarding nursing practices for patients with CI, particularly care processes relating to delirium prevention and management. The tool was informed by key Australian clinical guides [15, 16], and a chart abstraction tool developed for use in the Emergency Department [17]. Key items included in the tool are listed in Table 3. While nurse interactions with patients were directly observed and recorded, no data identifying any patient or nurse was recorded.

**Table 3:** Key items included within the Chart audit/ observational tool^ac^

Chart/ Room audit items	Response
Cognition	
Was the patient’s cognitive functioning assessed using a standardized assessment tool (e.g. MSQ, MMSE) within 24 h of the patient’s admission to the ward?	Yes/ No
Was the patient’s cognitive functioning assessed informally (i.e. not using a standardised assessment tool). Comments may include ‘oriented to person, time, place’ or ‘memory OK etc.’	Yes/ No
Pain Assessment and Management	
Was a pain assessment undertaken within the last 24 h/ since the last observation?	Yes/ No
Was analgesia administered within the last 24 h/ since the last observation?	Yes/ No
Medication Use	
Was antipsychotic medication administered to the patient within the last 24 h/ since the last observation?	Yes/ No
Was benzodiazepine medication administered to the patient within the last 24 h/ since the last observation?	Yes/ No
Items requiring direct observation	Response
Behavior	
Did the patient display symptoms of agitation (e.g. moaning, calling out, pacing, fidgeting, hand wringing etc.) OR pain OR discomfort (e.g. frowning, grimacing, holding onto any part of his/ her body, crying or moaning etc.)?	Yes/ No/ N/A^b^
What was the patient doing at the time of the observation?	Various, e.g. Asleep/ Lying in bed – no activity – engaged in activity / Sitting in chair
Orientation	
Did the nurse address the patient by name when he/ she interacted with the patient?	Yes/ No / N/A
Did the nurse introduce themselves when they interacted with the patient?	Yes/ No/ N/A
Did the nurse re-orient the patient if confusion/dis-orientation was evident?	Yes/ No / N/A
Communication	
Did the nurse explain the activity/ procedure in easy-to-understand terms to the patient?	Yes/ No / N/A
Nutrition Was adequate assistance provided to the patient if the patient had difficulty eating or drinking during meal-times?	Yes/ No / N/A

^a^Examples of each category are included for illustrative purposes

^b^N/A – Not Applicable - either the patient or nurse was not present during the observation period

^c^A number of additional items were included in the audit tool (e.g. Did the patient had an indwelling catheter in situ? Had a restraint order been written for the patient?), however the incidence was very low (there were nil restraint orders) and have not been included in this report as they were considered uninformative

### Data collection

Three Research Nurses collected the data at both sites, following training to use the Audit/ Observational tool. Inter-rater reliability was assessed by the nurses and CT independently, but simultaneously conducting a live audit on one hospital patient (not included in the results), and results indicated reliability to be very high (kappa = 1.0). Audits/ observations were undertaken for one full day in each of the six IH and two CH wards on four occasions corresponding to key project activities: Baseline (pre-intervention), Data Collection 2 (immediately following the CogChamps’ education), Data Collection 3 (Post Action Plan implementation), and at Follow-Up (three months following withdrawal of the Research team) (see Table 2). On the morning of each audit, the Research Manager (CT) identified all patients aged 65 years and older with a documented diagnosis of dementia or delirium, or report of confusion, memory problems or other CI in the patient’s chart, or reported verbally by the Charge Nurse. A maximum of eight patients were selected for audit/ observation on four separate, regularly spaced occasions throughout the day. Patients were excluded only if they were likely to be absent from the ward for much of the day (e.g. if a procedure had been scheduled), or if there were more than eight eligible patients on any one day, in which case a randomization procedure was used (see [12] for details).

### Ethics

Ethics approvals were obtained from the Human Research Ethics Committees of the Queensland University of Technology and Metro-South Health, which is responsible for the participating hospitals. As no personally identifying information was recorded for either nurse or patient, obtaining individual informed consent from each participant was not required by the relevant Ethics Committees.

### Education and empowerment of champions

The Facilitator CogChamps undertook a very active role in working with the other CogChamps to assist them to progress their Action Plans. They provided direct support by conducting education sessions, providing bed-side teaching and role-modelling best practices, sourcing resources and maintaining records. These Facilitator CogChamps were highly credible as change agents, being very experienced RNs who had worked on their home wards for several years and who were well known and respected by local ward staff. Furthermore, they had a contemporary understanding of the hospital’s workplace culture including its processes and practices. The placement of the Facilitator CogChamps on the wards in an “off-line capacity” (i.e. no direct patient allocation) meant they were readily available to support other CogChamps and coach and mentor ward nurses when opportune moments for teaching and modelling best-practices arose (e.g. when a patient with CI required nursing care). Examples of the activities undertaken by the facilitators are summarized in Table 4.

**Table 4:** Activities undertaken by the Facilitator CogChamps

Education	
Conduct small group delirium education sessions for nurses;	
Provide 1:1 education at the bedside about delirium – its features and risk factors, and prevention and management strategies;	
Educate nurses in the correct administration and interpretation of the short Confusion Assessment Method (CAM), [18, 19] to assess patients for delirium;	
Educate nurses regarding the correct recording of a completed CAM assessment in the hospital’s electronic medical records system;	
Provide education regarding appropriate interventions (including referral to medical staff) to manage patients with delirium;	
Role model best practice care approaches to nurses e.g. how to re-orient a patient who appears confused, how to communicate with a patient with CI, how to calm a distressed patient, how to engage patients in meaningful activity and how to assess pain;	
Prompt discussion of delirium as a vital sign at hand-over;	
Promote staff awareness of Delirium checklists (e.g. PITCHED – see below) [22];	
Assist staff in identifying modifiable triggers to behavioral symptoms in patients with CI;	
Support	
Assist nurses to provide direct patient care for patients with CI and demonstrate useful management strategies;	
Assist nurses develop appropriate care plans for patients with CI;	
Assist nurses contact patient’s families/ relatives to gather important information regarding the patient’s care preferences in order to provide person-centered care;	
Assist CogChamps collect data for evaluation purposes (e.g. mini-audits of CAM use were conducted on two wards prior to and following the implementation of Action plans to assess whether any change had occurred);	
Assist the CogChamps to access evidence-based resources to support patient care e.g. contacting hospital medical illustration unit to develop lanyards for the PITCHED tool [22];	

### Action plans

All six Action Plans included items that focused on (a) improving nurses’ knowledge about dementia and delirium and how to identify delirium, (b) increasing nurses’ assessments of at-risk patients for delirium at admission to the ward, and (c) improving the nursing care of patients with CI. Four wards also identified the need to improve how staff communicated with each other about patients with CI, and was also included as an action item in their plans. Details of the educational activities undertaken [i.e. Action Plan items (a) and (b)] together with associated outcomes have been reported elsewhere [14]. Briefly, those activities resulted in the education of a majority of all ward nurses about delirium (70%) and its identification using the short CAM (77%) [18, 19], across the six IH wards. Some CogChamps also performed mini-audits of the rates of cognitive assessments performed by nurses to provide feedback regarding progress towards this Action Item. These data were collected by the CogChamps and provided evidence of an increase in the number of at-risk patients assessed for delirium early in their hospital admission following the education. Details of the additional Action Plan items implemented are summarized in Table 5.

**Table 5:**
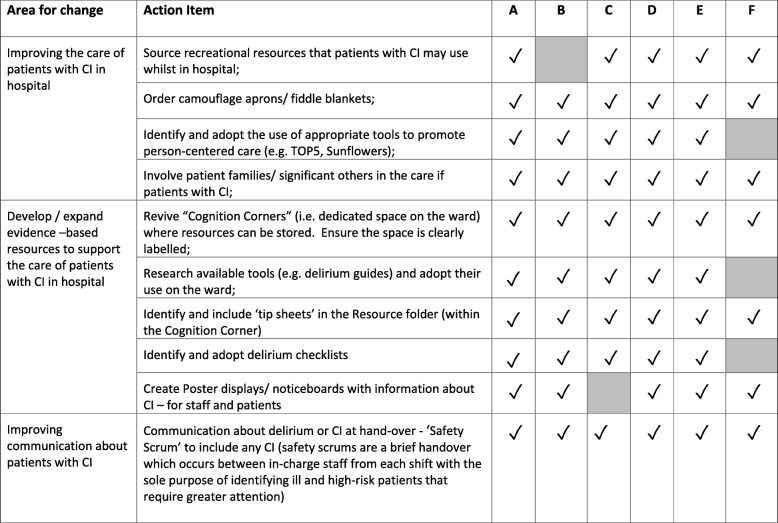
Summary of Action items to improve the care of patients with cognitive impairment^ab^

^a^Shading indicates activity not undertaken; ^b^ Action Plan items regarding nurse education are provided in the companion paper [14]

### Implementation of different recreational, social and clinical resources

All wards developed or acquired resources to assist nurses and/or patients and their families when caring for patients with CI. These included the revitalization of the Cognition Corners in each ward so that more resources were available to engage patients with CI in meaningful recreational and social activities (e.g. playing cards, simple games such as ‘Connect 4”, magazines), the inclusion of resources to support nurses when caring for patients with CI (e.g. ‘12 Top tips in caring for a person with dementia’) [20], and resources to support patients with CI while in hospital (e.g. ordering decoy-camouflage aprons which are made by hospital volunteers for use by patients with more severe CI who might injure themselves by pulling out their indwelling catheter or other device). Four wards developed poster displays that included information about delirium and dementia for patients, families and staff.

### Implementation of clinical tools

Three wards chose to use the acronym PITCHED to assist them when nursing patients with delirium Similar to a UK tool known as PINCH-ME [21], the PITCHED acronym reminds staff to monitor for Pain, Infection, Thirst-Hydration, Constipation, Hunger-Nutrition, Environment, Drugs in patients with or at-risk of delirium [22]. The acronym was promoted in various ways including discussion at ward meetings, and by providing lanyards of PITCHED with its meaning printed and distributed to all nurses on those wards.

One ward selected the TOP5 resource to assist nurses to provide personalized care for patients with CI [23]. The tool encourages hospital staff to talk to the patient’s relative or significant other to obtain important information about the person with CI so that their care may be personalized and strategies identified that may assist if the person becomes anxious or distressed.

### Other strategies

CogChamps from all wards developed and/or acquired a range of resources to improve the care of patients with CI when hospitalized. These included posters, handouts for patients and families, the adoption of checklists to assist nurses to care for patients with delirium as well as resources for patients to use while in hospital. The latter included recreational resources for patients (e.g. card games), and camouflage aprons/ fiddle blankets.

While improving communication amongst clinical staff about patients with CI was specified as an Action Item, because communication about patients with CI occurred at the bedside CogChamps were not asked to collect data regarding this item as it was considered onerous to do so. Hence, we do not have accurate data regarding this item, although self-report data from two CogChamps indicated that communication about CI had increased.

### Data analysis

Group differences were assessed using the Student’s *t*-test for continuous data, and non-parametric tests (Chi-squared statistic) for categorical data. Insufficient data were available for several outcomes at the CH for some time-points [e.g. number of cognitive assessments ranged from 1 (10%) to 4 (50%) at the CH], precluding analysis of change across time; hence these data were collapsed across all time-points (Baseline, Data Collections 2 and 3 and Follow-Up) in order to compare the two hospitals on these outcomes (see Table 6). All analyses were performed using SPSS for Windows version 21.0 [24].

**Table 6:** Observational Outcome Data - observations across all time-points^c^

	Intervention Hospital	Control Hospital	χ^2^	P-value
PATIENT BEHAVIOR				
Patient activity:				
Patient doing nothing, versus	170 (45%)	81 (52%)	1.99	0.16
Patient engaged in some activity	206 (55%)	75 (48%)		
(n; % of total observations)	Total obs = 376	Total obs = 156		
Patient showed signs of agitation/ pain (n; % of total observations)	59 (12%)	25 (14%)	0.69	0.41
	Total obs = 497	Total obs = 175		
Patient showed confusion/ disorientation (n; % of total observations)	184 (38%)	74 (38%)	0.012	0.91
	Total obs = 484	Total obs = 197		
Number of occasions, patients experienced meal-time difficulty (n; % of total observations)	128 (62%)	55 (70%)	1.28	0.27
	Total obs = 205	Total obs = 70		
NURSE BEHAVIOR				
Number of occasions patients who showed confusion were reoriented by nurse (n; % of total observations)	109 (63%)	36 (58%)	0.47	0.49
	Total obs = 173	Total obs = 62		
Number of occasions patients who experienced meal-time difficulty	96 (76%)	47 (87%)	3.0	0.56
received appropriate assistance^a^(n; % of total observations)	Total obs = 127	Total obs = 54		
How often did the nurse introduce themselves when they approached the	39 (10%)	34 (25%)	6.8	0.009*
patient (n; % of total observations)	Total obs = 384	Total obs = 135		
How often, did the nurse address the patient by his/her name when they approached the patient (n; % of total observations)	221 (57%)	67 (49%)	2.35	0.13
	Total obs = 386	Total obs = 135		
Nurse explained action/ procedure in easy to understand terms (n; % of total observations)	283 (71%)	97 (73%)	0.16	0.69
	Total obs = 398	Total obs = 133		
	Intervention Hospital	Control Hospital	χ^2^	P-value
	Total patients = 130	Total patients = 51		
Number of patients who received a newly prescribed antipsychotic within the last 24 h (n; % of patients)	29 (21%)	12 (23%)	0.03	0.86
Number of patients who received a newly prescribed benzodiazepine within the last 24 h (n; % of patients)	21^b^ (16%)	3 (6%)	3.36	0.07
Number of patients who were administered analgesia (n; % of patients)	36 (28%)	25 (49%)	7.46	0.006*
Assessment of patient’s cognitive function within 24 h of admission using a standardized assessment tool? (n; % of patients)	70 (54%)	13 (25%)	11.86	0.001**
Informal assessment of patient’s cognitive function (n; % of patients)	88 (68%)	31 (61%)	0.78	0.38

^a^Appropriate assistance was provided by nurses on a majority of occasions (91%); Others who provided assistance included family members /other staff

^b^One patient had experienced a seizure and two were undergoing alcohol/ drug detoxification

^c^The data presented in this Table have been collapsed across all time-points (Baseline, Data Collections 2 and 3, and Follow-Up); The number of observations differs as cases where the event did not occur (e.g. there was no nurse – patient interaction during the observation) were excluded from the analyses

^*^p < 0.05; **p < 0.001

## Results

### CogChamps

Thirty-four CogChamps participated in the intervention, of which the majority were female (84%), aged between 21 and 40 years (84%) with three or more years of nursing experience (71%).

### Participants

A total of 206 patients met the inclusion criteria across all data collection occasions. Fifteen patients were excluded as they were due for either discharge or surgery early in the day, and seven were randomized out as there were more than eight eligible patients on the ward on the one day. In addition, although not a specific criterion for exclusion, three patients who were actively dying were excluded out of respect for the families concerned. Thus, 181 patients were observed altogether: 130 patients at the IH, resulting in 500 discrete observations and 51 patients at the CH, resulting in 207 observations. Important demographic characteristics of the patients at baseline are presented in Table 7. While patients at the CH were significantly older than patients at the IH (*p* < 0.01), there were no other significant differences between the two patient groups in terms of important demographic characteristics that were collected.

**Table 7:** Patient characteristics at the Intervention and Control hospitals

Characteristic	Intervention Hospital (*n* = 130); n (%)	Control Hospital (*n* = 51); n (%)	*P*-value
Gender Female	55 (42.3)	25 (49.0)	0.41
Male	75 (57.7)	26 (51.0)	
Age – Average (years)	80.40 (SD = 7.9)	84.51 (SD = 6.9)	0.001*
Range (years)	65–97	65–95	
Condition			
Dementia	40 (30.8)	15 (29.4)	0.74
Delirium	20 (15.4)	(11.8)	
Confusion	18 (13.8)	5(9.8)	
Memory Problems/ CI	28 (21.5)	13(25.5)	
Dementia/ CI & Delirium	22 (16.9)	12 (23.5)	
Acute Confusion due to organic	2 (1.5)	0	
cause (e.g. stroke, brain lesion)			
Medical ward patients	108 (83)	38 (74.5)	0.19
Surgical ward patients	22 (17)	13 (25.5)	

*P < 0.01

### Room & Chart Audit/ observation results

Results of the observational data collections, collapsed across all data collections, showing key patient and nurse behaviors, are summarized in Table 6. The data show that patients displayed signs of agitation/ pain around 12% of the time (e.g. shouting, grimacing, moaning and fidgeting), displayed confusion/ disorientation almost 40% of the time and experienced meal-time difficulties (e.g. difficulty opening containers, accessing cutlery) on a majority of occasions (IH: 62%; CH: 70%). These behaviors did not differ between the two hospitals, indicating the patient cohorts were very similar. Importantly, patients received appropriate assistance on most occasions that meal-time difficulties were experienced (76% of occasions), were re-oriented by a nurse around 60% of the time although around one-fifth of patients received a newly prescribed antipsychotic within the last 24 h (IH: 21%; CH: 23%). There was no change in these behaviors at the IH following the intervention. Few nurse behaviors differed between the two hospitals overall, except for the frequency of nurses introducing themselves when interacting with a patient, which was significantly higher at the CH compared to the IH (*p* = 0.009). Analgesia use was also significantly higher at the CH while the assessment of patients’ cognitive function was significantly higher at the IH.

### Outcomes following the CogChamps intervention

#### Assessment of patient’s cognitive functioning within 24 h of admission

Insufficient cognitive assessments had been performed at the CH for data analysis and hence, only data for the IH were analyzed to determine whether there had been a change in the number of cognitive assessments using a standardized tool within 24 h of admission to the ward following Action Plan implementation. Results showed there had been a significant overall increase in these assessments (χ^2^ = 12.00; *p* = 0.02) from baseline (35%) to post-implementation (69%) which was maintained at follow-up (72%). The increase was largely due to increased nurse activity, who performed significantly more assessments post-implementation (35%), compared to baseline (8%), maintaining the improvement at follow-up (44%; χ^2^ = 9.65, *p* = 0.05). Similarly, there was an increase in the proportion of patients whose cognitive functioning was informally assessed (i.e. charts included entries such as ‘patient was confused’ ‘patient was oriented to person, time and place’), that increased from 54% at baseline to 93% at Data Collection 4 (χ^2^ = 14.78, *p* = 0.005). but which declined to 76% at follow-up. While at a ward level there was variation in how much change occurred (see [12]), the data did not demonstrate any differences associated to whether the ward was medical or surgical. While wards varied in some of the resources they adopted, these were not liked to any significant changes or variation between wards.

#### Medication use at the intervention hospital

Insufficient medication use data were available at the CH for analysis, and hence and hence these data were analyzed for the IH alone. Results indicated a significant increase in the analgesia administration over time (χ^2^ = 13.15, *p* = 0.01), increasing from 27% at baseline to 48% post—Action Plan implementation, although subsequently declining to 12% at follow-up. Similarly, there was a significant change in benzodiazepine administration at the IH following the intervention (χ^2^ = 6.78, *p* = 0.03), decreasing from 24% at baseline to 6% post-implementation, although increasing to 20% at follow-up. By comparison, there was no change in the number of patients at the IH who received antipsychotic medication (χ^2^ = 2.34, *p* = 0.67) over time.

## Discussion

This paper describes the implementation and outcomes of a collective social education intervention designed to improve nurses approaches to caring for hospitalized patients with CI by increasing their adoption of multi-component non-pharmacological interventions. Cognition Champions (CogChamps) were supported to lead practice change efforts across six acute hospital wards by implementing tailored Action Plans that they had developed. Key outcomes included the education and upskilling of a majority of ward nurses about delirium and its assessment (see [14]), and significant increases in the proportion of patients whose cognitive functioning was assessed, both formally using a standardized assessment tool as well as informally. These findings suggest the project was effective in increasing nurses’ overall awareness of CI, particularly delirium, across the six wards. Improving the knowledge of hospital staff about CI and increasing the number of cognitive assessments performed have both been identified as key to improving the care of older patients with dementia when hospitalized [25, 26]. Thus, the project has achieved an important first step in improving the care of hospitalized patients with CI.

Other achievements include the development and/or acquisition of resources (e.g. checklists) to support nurses to provide better quality care for patients with CI. Furthermore, these resources were still being used at follow-up. There was, however, limited evidence of other practice improvements, with the exception of analgesia and benzodiazepine use. The administration of analgesia increased significantly while there was a significant decrease in benzodiazepine use at the IH following implementation. These findings suggest a practice improvement given that pain in patients with CI is frequently under-treated [27], and adequate pain relief and avoiding benzodiazepine use are important strategies for preventing delirium [16], while benzodiazepines should be avoided in patients with CI [28]. While our results indicate there may have been an increase in nurses’ awareness of pain and its treatment as important for patients with CI, particularly as a risk factor for delirium [5]. However, our data does not indicate whether analgesic use was optimal (i.e. administered at the right time for the right reasons). Interestingly, pre-intervention benzodiazepine use was considerably higher and analgesic use was significantly lower (overall) at the IH than at the CH even though the patient cohorts were very similar. The reasons for this difference are unknown.

In summary, the project’s key achievements included the education of a majority of ward nurses about delirium and its assessment using the CAM, and an increase in cognitive assessments. Both were explicitly identified as Action items in the Action Plans [14], with associated targets explicitly outlined (e.g. 80% of ward nurses would receive education), and specific CogChamps assigned to maintaining attendance records and evaluating progress. By comparison, we found limited evidence of practice change when activities were less explicitly defined and could be conceived as discretionary. For example, we found no improvements in nurses re-orienting confused patients (confusion was evident in 38% of observations) or in providing meal-time assistance to those who needed it (a majority of patients at both hospitals), although it is possible that our audit/observational tool lacked sensitivity to detect changes in these outcomes. It is encouraging, however, to note these actions were implemented most of the time prior to the intervention indicating reasonable awareness of patient’s needs and ways to address them. However, it also means some patients may have received inadequate nutrition or may have remained confused for longer than necessary, both of which may have increased their susceptibility to adverse events including delirium [21, 25]. In comparison to the educational activities, these action items were less explicitly defined in the Action Plans (e.g. ‘adopt delirium checklists’), and the selection of a specific tool or resource for use on the ward was determined by the ward’s CogChamps.

While all wards adopted resources to improve the care of patients with CI and promoted their use for patients with CI, use of the resource or tool by individual nurses was discretionary (i.e. up to the individual nurse). CogChamps were not required to maintain records auditing resources use, as this would have been impractical. The omission of care tasks that are viewed as everyone’s responsibility but for which no one is directly accountable has been previously reported to account, at least in part, for poor nutritional intake in older medical inpatients [29]. Organizational factors associated with efficiency and risk have been linked to how nurses ration their care tasks, so that only essential or emergency care tasks are prioritized and other “discretionary” tasks, while significant to the health and well-being of older confused patients, are often never completed [30]. The non-completion of care tasks due to no staff member assuming responsibility for the task was also reported by Mudge and colleagues in their multi-component, multi-disciplinary intervention to improve hospital outcomes for older patients [31]. Although they addressed the issue by employing an allied health assistant to assume responsibility for these discretionary tasks, this solution is unlikely to be feasible in many instances due to funding issues, and other approaches are needed which will require sound clinical leadership. Indeed, a panel of delirium experts has recently concluded that delirium will continue to be under-recognized and under-treated without a concerted effort by hospital leaders to increase the priority it is accorded [10].

### Barriers and enablers

The project faced the usual well-documented challenges associated with the implementation of practice change in the busy hospital environment including heavy workloads, competing demands, the complexity of care required and the care environment itself, the possibility that nurses may not be committed to the project as well as the challenges associated with shift work and attrition e.g. [32–34]. The secondment of the CogChamps to work as facilitators on the project, however, effectively overcame some of the time constraints and enabled progression of the Action Plans. The placement of the facilitators on the wards meant they were readily available to support the CogChamps and coach and mentor nurses at times that were convenient to them. Moreover, they were available to teach and model desired behaviors when opportune moments presented – a strategy identified as essential by a panel of delirium experts [10].

In this study, the facilitators reported modelling was a very effective tool for promoting the adoption of new behaviors, particularly when nurses could see direct benefits of the changed practice(s). Although the facilitators were novices in that role, they succeeded partly because of the mentorship they received from AH, but also because they possessed characteristics that have been reported to be important in a good facilitator [33]. These included the ability to communicate clearly, being tenacious (kept on going when some nurses showed disinterest), and being able to think creatively about patients and patient care and mentor nurses to do likewise.

Other facilitating factors included support from both the NUMs and the Research Team and the development of ward specific Action Plans, led by the CogChamps, ensuring each plan was tailored to address each ward’s specific requirements and circumstances. The Action Plans provided a concrete guide and clear direction for the practice improvement efforts and appears to have effectively engaged the CogChamps in the project. Development of the plans is also likely to have increased the CogChamps’ awareness of the need for change, which has been identified as important in motivating implementation efforts [33].

Finally, while we attempted to address project sustainability at commencement through, for example, encouraging CogChamps to embed changes into daily practice and recruit new CogChamps to address attrition, there was some evidence that the project’s impact waned following completion. For example, data collected at follow-up showed a decline in the use of analgesia compared to post-intervention although cognitive assessments remained improved at follow-up. This might be partly due to the introduction of another hospital wide initiative at the completion of CogChamps, but like any practice improvement project, the project’s impact is likely to continue to decline without continued emphasis on delirium as an important issue and addressing workplace and organizational barriers to delirium recognition, prevention and treatment [10].

### Study strengths and weaknesses

An important strength of this study is the collection of data by direct observational of nurse-patient interactions to provide direct evidence of practice change. The Research Nurses were trained to administer the tool accurately and we are confident the data accurately reflects nurse’s behaviors and interventions which are often poorly documented in medical charts [35].

One study limitation includes its relatively small-scale and although a large number of patient observations were conducted, data in relation to some outcomes were insufficient for data analysis (e.g. medication data at the CH), primarily due to a low event rate. Fewer observations conducted on a larger sample may have overcome this limitation. A second limitation is its implementation in one hospital only which had established a sound foundation for improving the care of patients with CI through a number of previous initiatives so had a level of readiness for staff practice change. The degree to which this ‘priming’ facilitated the current project is not known and hence, the transferability of the CogChamps project to other hospitals without this background and without this project’s resources, is not known. It should also be noted that because a range of quality improvement activities had been implemented in the IH over several years prior to this study, demonstrating significant changes in care practices through only targeting local leadership and individual staff training and knowledge may have been more difficult to attain. A recent qualitative study about practice gaps in delirium care suggested that organizational factors may substantially impede the ability of individuals to provide evidence-based care to patients with CI even if they have the knowledge and will [36].

## Conclusion

The CogChamps project demonstrates that nurse Champions can be effectively empowered to educate other nurses about dementia and delirium including the accurate recognition of delirium. Practice improvements were achieved for well-defined tasks (e.g. conducting a cognitive assessment) with clear targets, the nomination of those responsible for the task and where progress was measured. Thus, it is concluded that these elements are important when implementing practice changes. Although only modest improvements were demonstrated, these results suggest that using collective social education approaches shows promise and warrants further research.

## Abbreviations

ACSQHC: Australian Commission on Safety and Quality in Health Care
CH: Control Hospital
CI: Cognitive Impairment
CogChamps: Cognition Champions
IH: Intervention Hospital
KT: Knowledge Translation
RN: Registered Nurse
